# Protein Complex Identification by Integrating Protein-Protein Interaction Evidence from Multiple Sources

**DOI:** 10.1371/journal.pone.0083841

**Published:** 2013-12-27

**Authors:** Bo Xu, Hongfei Lin, Yang Chen, Zhihao Yang, Hongfang Liu

**Affiliations:** 1 School of Computer Science and Technology, Dalian University of Technology, Dalian, China; 2 Department of Health Science Research, Mayo Clinic, Rochester, Minnesota, United States of America; 3 Department of Computer Science, Virginia Tech, Falls Church, Virginia, United States of America; MRC National Institute for Medical Research, United Kingdom

## Abstract

**Background:**

Understanding protein complexes is important for understanding the science of cellular organization and function. Many computational methods have been developed to identify protein complexes from experimentally obtained protein-protein interaction (PPI) networks. However, interaction information obtained experimentally can be unreliable and incomplete. Reconstructing these PPI networks with PPI evidences from other sources can improve protein complex identification.

**Results:**

We combined PPI information from 6 different sources and obtained a reconstructed PPI network for yeast through machine learning. Some popular protein complex identification methods were then applied to detect yeast protein complexes using the new PPI networks. Our evaluation indicates that protein complex identification algorithms using the reconstructed PPI network significantly outperform ones on experimentally verified PPI networks.

**Conclusions:**

We conclude that incorporating PPI information from other sources can improve the effectiveness of protein complex identification.

## Introduction

A protein complex is a group of associated polypeptide chains linked by noncovalent protein-protein interactions (PPIs). Protein complexes have an important role in biological processes and perform independent discrete biological functions, such as DNA transcription, mRNA translation, and signal transduction [1]. Hence, identifying protein complexes in an organism is critical in molecular biology. Protein complexes can be identified with high accuracy using small-scale experimental techniques such as immunoprecipitation, but such techniques are time-consuming and tedious [2]. Recently, several high-throughput methods have been used to detect PPIs on a larger scale, including the yeast 2-hybrid system, mass spectrometry, and protein chips.

Computational approaches also can be applied to identify protein complex information by searching densely connected regions in a PPI network [3], a graphical map of an entire organism’s interactome. This is constructed from existing PPI knowledge by considering individual proteins as nodes and the existence of a physical interaction between a pair of proteins as a link. The existing PPI knowledge, however, is generally built using information gathered with the high-throughput techniques mentioned above, which can be unreliable and incomplete [4]. Therefore, many recent studies have tried to combine PPI information from multiple sources to improve the accuracy of the PPI information. For example, a graph fragmentation algorithm incorporated microarray gene expression profiles to help refine the putative complexes [5]. With this method, the running time is proportional to the number of samples and could become a concern if the PPI network is large. Jung et al [6] presented a simultaneous protein interaction network, which deleted any mutually exclusive interactions based on domain information. Ozawa et al [7] also considered the competition between mutually exclusive interactions. They accounted for the structural limitations of the proteins and determined whether the proteins in the extracted complex could simultaneously bind to each other. Xu et al [8] weighted PPI networks on the basis of the semantic similarity of each protein pair in the Gene Ontology project (GO). CMC (clustering based on maximal cliques) [9] used an iterative scoring method to assign a weight to protein pairs, which indicated the reliability of the interaction between the 2 proteins. Krogan et al [10] used high-throughput purification data to predict protein complexes.

In the current paper, instead of using 1 or 2 sources to predict protein complexes, we applied machine learning to predict PPI pairs from 6 diverse sources and supplemented the reliable PPIs with predicted PPI pairs. Since protein structures provided a strong evidence for Protein-Protein Interactions prediction, we selected positive PPIs based on Domain-Domain interaction (DDI) information and purified PPI datasets Krogan core [10] and Collins [11]. 12K reliable positive PPIs are obtained for training which are from Krogan, Collins and have DDI. However, it is rare to find confirmed reports of *noninteracting* pairs, especially not on a large scale. Hence, learning from positive and unlabeled data (LPU) [12], [13] is a good way to handle this problem. To increase the reliability of predictions, an ensemble approach can be used whereby predictions from multiple LPU classifiers are obtained by alternating the number of unlabeled instances. We evaluated the ability of our method to predict yeast protein complexes. Protein pairs were represented using 18 features gathered from 6 sources. We obtained a predicted PPI network through LPU whereby a protein pair was considered to be positive if it is recorded in Krogan core dataset, Collins and has reliable DDI evidence. All other protein pairs were treated as unlabeled. We then built 5 LPU classifiers and chose the top *n* pairs as the predicted PPI pairs. Some popular protein complex identification algorithms COACH (a core-attachment method) [14], CMC [9], CFinder [15], MCODE [16], IPCA [17], MCL [18] and Clusterone [19] were then applied on a reconstructed PPI network built upon reliable PPIs and predicted PPI pairs. The data and algorithms are available in our supporting website: http://202.118.75.18∶8080/PPINPredictor/. We also evaluated our LPU model and compared the performances on reconstructed PPI network with ones on the DIP [20], Krogan [10], BioGRID [21] PPI networks.

## Methods

**Figure 1:**
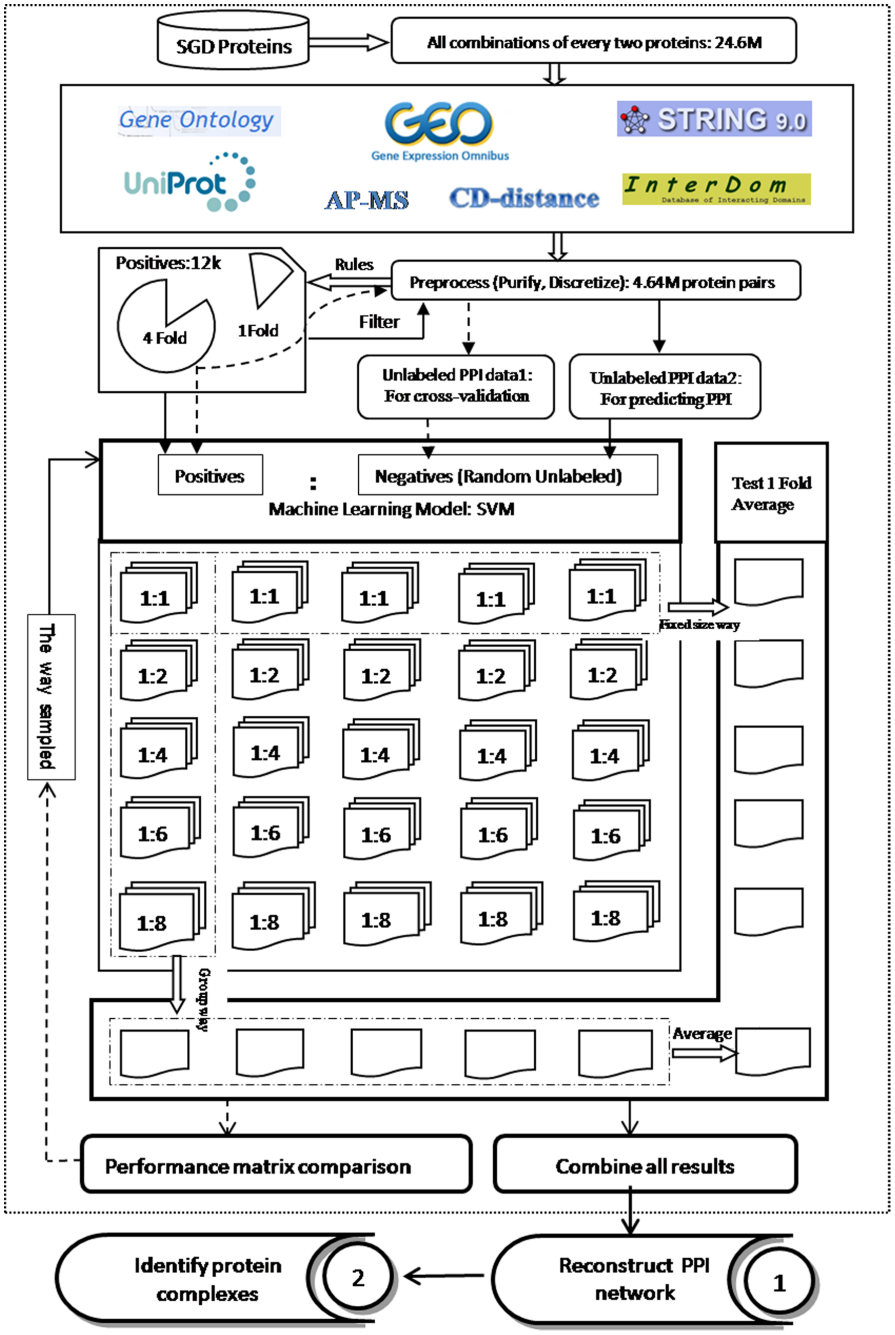
Flowchart of experimental method. There are 2 main steps in the method. The first is to rank protein pairs according to their probabilities or confidence scores of being true protein-protein interaction (PPI) pairs by defining a machine learning task. The second is to apply the state-of-the-art protein complex detection algorithm to a reconstructed PPI network. In the first part, we represented protein pairs based on 6 sources, then used learning from positive and unlabeled data (LPU) to predict PPIs. We also applied a five fold cross-validation for evaluating the LPU model. In the second part, we applied some popular protein complexes detection methods (COACH, CMC, CFinder, MCODE, IPCA, MCL, Clusterone) on a reconstructed PPI network to identify protein complexes.

For a given organism, the proposed protein complex identification approach contains 2 steps (Figure 1). The first step is to rank protein pairs according to their probabilities or confidence scores of being true PPI pairs by defining a machine learning task. The second step is to apply the state-of-the-art protein complex detection algorithms, but the PPI network is reconstructed with reliable PPI and predicted PPI pairs. Here, we first describe features considered to be PPI related and then present the detailed LPU approach for ranking protein pairs. We then introduce 7 state-of-the-art protein complex detection algorithms.

### Sources with PPI Evidence

The following sources are considered PPI-related features.

#### Gene ontology annotations

GO [22] contains 3 hierarchies that hold terms defining the basic concepts of molecular function (MF), biological processes (BP), and cellular components (CC), respectively. GO terms are arranged in directed acyclic graphs. Several GO Slims (ie, slim versions of GO) have been defined, in which each contains several dozen high-level GO terms. If two proteins have interaction relation, they always participate in the same biology process or happened in the same cellular component, some of them even have similar functions. So a protein pair with similar GO annotations has a higher probability of being a PPI pair. We used 2 different types of measures to calculate the similarity of GO annotations for a protein pair. One type (Type I) is based on organism-specific GO Slims. If 2 proteins in a pair shared at least 1 common GO Slim term after removing trivial root GO terms, we assigned a similarity value of 1; otherwise, the value was 0. The other type (Type II) uses the semantic similarity measure of Lord et al [23]. It is based on the hypothesis that a term is more informative if it and its descendants have fewer annotated genes or proteins in an ontology. For instance, ‘chaperone’, (GO:0003754) is a more informative term than ‘signal transducer’, (GO:0004871), because the former is used several hundred times, while the latter is used several thousand times. The similarity of GO annotation measure starts with a probability measure of each term *t.* Let *D_t_* be the collection of GO terms that are either *t* or its descendants. Let *A(t, c)* be the occurrence of *t* annotations given a collection *c*. The probability of *t* in *c*, or *p(t, c)*, is defined as:
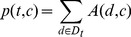
(1)


Let *CA(t_1_, t_2_)* be the lowest common ancestor set for terms *t_1_* and *t_2_*, since GO allows multiple parents for each term. The semantic similarity of two GO terms is defined as:
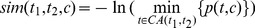
(2)


The similarity of two genes or gene products is then defined as the highest similarity between GO annotations for them. Here we calculate BP, CC, MF similarity separately as three features. A total of 6 features were defined by combining the 2 similarity types and the 3 hierarchies.

#### Gene coexpression

The corresponding genes of the proteins in a protein complex are expected to be coexpressed (ie, activated and repressed under the same conditions) [24]–[26]. We defined a feature to capture gene coexpression information of a protein pair by using many microarray data series available in Gene Expression Omnibus [27]. The value was set to be the Pearson correlation coefficient of the 2 genes in those series.

#### Domain-Domain interaction

**Figure 2:**
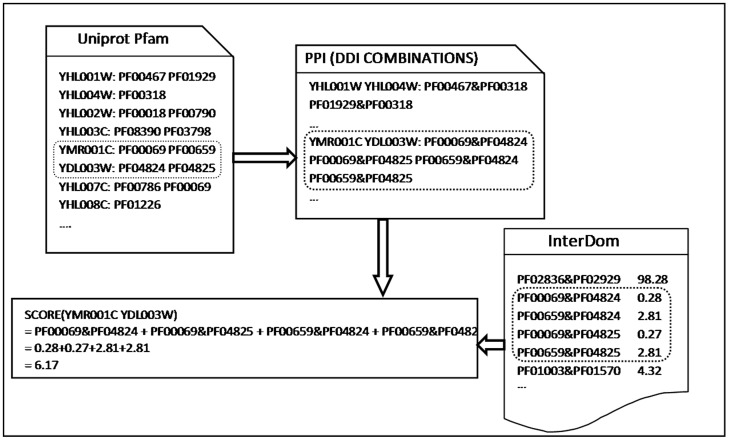
The score of Domain-Domain interaction for each protein pair. Calculating DDI value based on UniprotKB and InterDom datasets.

A protein domain is a conserved part of a given protein sequence and structure that can evolve, function, and exist independently of the rest of the protein chain. Domains often suggest the propensity for the proteins to interact or form a functional unit, such as protein complex. If two proteins have more domain-domain interactions (DDI), they have more possibility to have an interaction. So we used one feature to capture DDI information for a protein pair. As shown in Figure 2, we retrieved protein domain information from UniprotKB [28] with query taxonomy 4932, if one protein has domain information, it is represented by domain list, such as protein YMR001C. We also downloaded DDI information from InterDom [29], a putative domain-domain interaction (DDI) database, in which each DDI pair is assigned a confidence score. So protein pairs can be represented by DDI pairs, such as PPI YMR001C and YDL003W in Figure 2. For each protein pair, we calculated the sum of the confidence scores of all possible DDI pairs as a DDI feature value.

#### STRING evidence

STRING [30] is a database of direct (physical) and indirect (functional) protein interactions. These known and predicted protein interactions are derived from 4 sources: genomic context, high-throughput experiments, coexpression (conserved), and prior knowledge. We consider it to be an essential source, since it contains PPI information extracted from the literature and several other sources. A score is assigned by STRING for each protein pair to indicate the confidence of PPI. We used that score as the feature to capture STRING-predicted evidence of PPI information.

#### AP-MS experiments

Affinity purification combined with mass spectrometry (AP-MS) is a powerful method for high-throughput PPI identification. Affinity purification consists of first tagging a protein of interest (bait) by genetically inserting a small peptide sequence (tag) onto the recombinant bait protein. The bait protein is affinity purified, together with its interacting partners (preys), which are identified using mass spectrometry. The partners can have either a direct physical interaction with the bait or an indirect physical interaction mediated by a protein complex. AP-MS experiments provide direct information about co-complex relationships among proteins. However, proteins in the same protein complex may be missed in the screen (false-negatives) if they fail to bind tightly enough, whereas other proteins may be copurified if they bind nonspecifically to the bait (false-positives). Because of these false-negatives, false-positives and the datasets are large, computational methods have been developed to isolate true protein complexes out of the purification results. These computational methods typically convert the co-complex relationships in the AP-MS data into binary PPIs. They proposed different measurements to assigns a reliability score to every protein pair in converting multirelationships into binary interactions, such as socio-affinity index by Gavin [31]. It is based on the log-odds of the number of times two proteins were observed together in a purification, relative to the expected frequency of co-occurrence of two proteins in purifications. The higher the score are, the more reliable of the candidate PPIs. Here we downloaded the candidate PPIs with reliable score from Gavin [31], Krogan [10], Collins [11] and Hart [32], each reliability measurements result can be defined a AP-MS feature [10], [32]–[34].

#### PPI network properties

Not every interaction pair is present in curated PPI networks. We consider a protein pair to have a higher probability of being a PPI pair if they have many common neighbors in a PPI network. We use the Czekanowski-Dice distance (CD-distance) to capture such information. Given a pair of proteins X and Y in an interaction graph G, *CD-Dist^G^(X,Y)* is defined as the proportion of partners that the 2 proteins have in common:
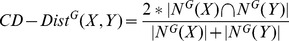
(3)where N^G^(X) and N^G^(Y) are the set of neighbors for X and Y. CD-distance, originally proposed by Brun et al [35] to predict function, was later shown to effectively assess the reliability of high-throughput interaction data [36].

### Learning from Positive and Unlabeled Data

Using machine learning to predict a protein pair to be a PPI pair (or not) requires a training set containing pairs that are annotated as positive or negative. The positive pairs can be obtained from curated knowledge sources with protein structure evidence, whereas a confirmed report of noninteracting pairs is difficult to obtain. We randomly selected unlabeled protein pairs to act as negative pairs, since only one in several hundred potential protein pairs actually contain interacting partners. Thus, over 99% of our random data is indeed noninteracting, which is probably better than the accuracy of most training data. This randomly sampling negatives way is popular applied in LPU (Learning from positive and unlabeled data) model for many researches and got a good performance, such as ref. [37], ref. [12] and ref. [13]. So LPU (Learning from positive and unlabeled data) model can be applied for our task. The class distribution in the training set can affect the performance of the resulting systems, so we used the following strategy to generate multiple negative examples. For a given set of PPI pairs, multiple sets of negatives were sampled using different class distributions; the ratios of positives and negatives were set to be {1∶1, 1∶2, 1∶4, …, 1∶2*n}. We repeated the strategy *m* times and constructed *m*×(*n*+1) classifiers for a chosen machine learning algorithm. Each of the classifiers assigns a score to each of the unlabeled pairs, in which the score measures the possibility or confidence of a pair being positive. We then rank the unlabeled pairs by the sum of the *m*×(*n*+1) scores; those ranked high are considered to be *predicted PPI pairs*.

### Protein Complex Identification Algorithms

After acquiring predicted PPI pairs, existing computational methods developed to identify protein complexes from PPI networks can be used. We employed 7 state-of-the-art protein complex identification algorithms here: COACH [14], CMC [9], CFinder [15], MCODE [16], IPCA [17], Clusterone [19] and MCL [18].

COACH [14] is based on a core-attachment [38] method and detects protein complexes from PPI networks. It mines protein complex cores from neighborhood graphs and forms protein complexes by including attachments into cores. Proteins placed in the same protein complex core are functionally similar and tend to be colocalized [39].

CMC [9] finds complexes from the weighted PPI network based on maximal cliques. It first uses an iterative scoring method (AdjustCD) to assign weight to protein pairs. The weight of a protein pair indicates the reliability of the interaction between the 2 proteins. It then generates all the maximal cliques from the weighted PPI networks. It finally removes or merges highly overlapped clusters based on their interconnectivity to determine protein complexes.

Adamcsek et al. [15] provided a software called CFinder to find functional modules in PPI networks. CFinder detects the k-clique percolation clusters as functional modules using a Clique Percolation Method [40]. In particular, a k-clique is a clique with k nodes and two k-cliques are adjacent if they share (k –1) common nodes. A k-clique percolation cluster is then constructed by linking all the adjacent k-cliques as a bigger subgraph.

MCODE algorithm proposed by Bader et al. [16] is one of the first computational methods to detect protein complexes based on the proteins’ connectivity values in the PPI network. MCODE first weighs every node based on their local neighborhood densities, and then selects seed nodes with high weights as initial clusters and augments these clusters by outward traversing from the seeds. In addition, MCODE has an optional post-processing step with operations such as filtering non-dense subgraphs and generating overlapping clusters.

IPCA [17] is a modified DPClus [41] algorithm which expands clusters starting from seeded vertices. It per-forms a better performance than DPClus since it proposes a new topological structure for protein complexes, which is a combination of subgraph diameter (or average vertex distance) and subgraph density.

Clusterone [19] algorithm consists of three major steps (Online Methods). First, starting from a single seed vertex, a greedy procedure adds or removes vertices to find groups with high cohesiveness. In the second step, they quantify the extent of overlap between each pair of groups and merge those for which the overlap score [16] is above a specified threshold. In the third step, they discard complex candidates that contain less than three proteins or whose density is below a given threshold. Note that their method can detect potentially overlapping protein complexes.

MCL [18] (Markov Clustering) is a method that identify protein complexes by simulating random walks in PPI networks. It contains two steps: expansion and inflation. The expansion step assigns new probabilities for all pairs of nodes, while the inflation step changes the probabilities for all these walks in the graph. Iterative expansion and inflation will separate the PPI network into many parts as protein complexes.

## Experiments

### Performance Evaluation

We followed existing approaches [39], [42], [43] to evaluate the experimental performance. Equation 4 calculates the neighborhood affinity score *NA(p,b)* between a predicted cluster *p ∈P* and a real complex *b ∈B*, where *P* is the set of predicted complexes by a computational method and *B* is the set of real ones in the benchmark.
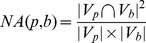
(4)


In equation 4, *|V_p_|* is the number of proteins in the predicted complexes and *|V_b_|* is the number of proteins in the real complex. If *NA(p,b)≥ω*, a real complex and a predicted complex are considered to be matching (ω is usually set as 0.20 or 0.25) [3].

After all real complexes and predicted clusters have their best match calculated according to their *NA* scores, precision, recall, and F-measure are applied to assess the methods:
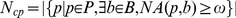
(5)

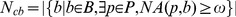
(6)

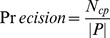
(7)

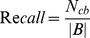
(8)

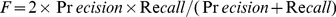
(9)


N_cp_ is the number of predicted complexes that match at least 1 real complex, and N_cb_ is the number of real complexes that match at least 1 predicted complex [3].

There are lots of negative protein-protein interactions in the real world, we prefer to obtain the candidate PPI with high probability to be true. Since the prediction scores of a classifier indicates the probability of being positive in descending order, we chose Lift [44] which measures how fast to obtain positive PPI. When ranking the results of a classifier based on its prediction score, the precision in top n is called estimated precision (EP). The baseline precision (BP) is the ratio of the number of positives over the total number of samples in the data set. The Lift is calculated using,
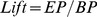
(10)which shows the relative utility of the classifier.

### Experiment Data

We evaluated our approach by performing a yeast protein complex identification task. We downloaded yeast protein interaction data from DIP [20], Krogan [10], BioGRID [21] PPI data for comparing the quality of our reconstructed PPI network. Krogan core [10] and Collins [11] datasets are also downloaded as candidate positive PPIs. We also retrieved 7,018 yeast proteins from the *Saccharomyces* Genome Database [45] and generated 24.6 million protein pairs. The yeast protein complex data were downloaded from a public repository (http://wodaklab.org/cyc2008/) with a total of 408 manually curated heteromeric protein complexes. After filtering out complexes composed of a single or a pair of proteins, the final benchmark set contains a total of 231 protein complexes.

The GO website was accessed in September 2011 to retrieve GO annotations and GO Slim terms for yeast. A total of 161 microarray data series for yeast (using platform PL90), consisting of 2,015 samples, were downloaded from Gene Expression Omnibus (accessed September 2011). The expression measures were log transformed, and a Pearson correlation coefficient was computed for each protein pair. We retrieved yeast protein domain information from UniProtKB [33] and DDI confidence scores from InterDom [46]. There are 7 computational results from AP-MS datasets for yeast [10], [32]–[34]; each assigns a reliability score to every protein pair in converting multirelationships into binary interactions. We used those scores directly as features.

### Evaluation of LPU Model

Because many protein pairs do not have PPI evidence from sources other than Gene Expression Omnibus and GO annotations, we used the following heuristics to filter out protein pairs:

1. There is only PPI evidence from Gene Expression Omnibus and GO annotation;
2. For the GO hierarchies BP and CC, Type I GO features have a value of 0 and Type II GO features have a value less than 0.002; and
3. The Pearson correlation coefficient is less than 0.5.

We obtained 4.64 million protein pairs according to the heuristics. Note that many of our features have numeric values. We applied discretization according to the value distribution (approximately equal frequency), which yielded a vector containing 281 elements for every pair.

Considering the protein structure is solid evidence, we integrate Domain-Domain interaction information for selecting reliable positive PPIs. As Krogan and Collins provided a purified PPIs and many methods utilized their datasets for protein complexes identification [19], we also selected candidate positive PPIs based on theirs. We considered the PPI as a reliable positive PPI which is recorded in Krogan core dataset, Collins dataset and its DDI value is above 0. We got 12477 reliable positive PPIs based on this rules. The unlabeled dataset is obtained by filtering out the positive PPIs from protein pairs datasets (Unlabeled PPI data 2 as shown in Figure 1). As mentioned above, it is difficult to find large amount of non-interact protein pairs and the proportion of positives to unlabeled is only one in several hundred. So LPU (Learn from positive and unlabeled data) is a proper way to deal this.

Considering class distribution in the training set can impact the performance of the resulting systems, so we used following two strategies to generate multiple negative examples. One we called group-way, another called fixed-size way. The group-way sampled multiple sets of negatives using different class distributions: the ratios of positives and negatives are set to be {1∶1, 1∶2, 1∶4, …, 1∶2n}. While the fixed-size way sampled them using same class distributions. We divided the positives into five folds and did a five-fold cross-validation for evaluating our LPU model. Each fold is selected as testing data in turn and the other 4 folds are for training. The testing fold is added to Unlabeled PPI data 2 for testing (called Unlabeled PPI data 1). Then we sampled 5 different size negative datasets based on Unlabeled PPI data 1. As shown in Figure 1, we got 25 different negative datasets for testing our model. Each row contained five same size negative datasets (fixed-size way) but five different testing dataset; each column contained five different size negative datasets (group-way) but same testing dataset. In order to find a proper way of generating negative datasets, we compared performances of these two ways by SVM. We calculated the average Lift value of each row and column separately as shown in Figure 1. For instance, the five models in first column gave five scores for each protein pair, we considered the sum of five scores as final score for each protein pair and got one Lift value. The average Lift value of five columns is calculated for comparison. While in the first row, the testing sets are different for five models, we got five Lift values for one fixed-size way and the average Lift value of five models is calculated for comparison.

### PPI Prediction

After evaluating the LPU, we selected group-way for predicting PPI. The parameters *m* and *n* in LPU were set to 5 and 4, respectively, with a total of 25 LPU classifiers constructed. The machine learning algorithm used was Support Vector Machine (SVM) implementation in libSVM 3.0. As shown in Figure 1, each of the classifiers assigns a score to each of the unlabeled pairs, in which the score measures the possibility or confidence of a pair to be positive. We then ranked the unlabeled pairs by the sum of the 25 scores from SVM, and those ranked high were considered to be predicted PPI pairs. Because the files are very large, the protein pairs with PPI evidence are shown in our supporting website (http://202.118.75.18∶8080/PPINPredictor/).

### Protein Complex Identification

We chose seven different popular methods to assess the performance of our methods. COACH, CMC, CFinder, Clusterone, MCODE, MCL and IPCA were implemented on the existed popular PPI networks and our new reconstructed networks respectively. We evaluated their performances on the Krogan, DIP and BioGRID PPI networks and compared them with our reconstructed PPI network built upon our purified reliable PPIs and predicted PPI pairs (12k+1000, 12k+2000, 12k+3000, 12k+4000 and 12k+5000).

## Results and Discussion

### Evaluation of LPU Model

For the fixed-size way (as shown in the Figure 1), we got five Lift values for each size. The average Lift values are calculated for comparison. For the group-way (as shown in the Figure 1), five results in the same column are added for each protein pair. The average Lift values of five columns are calculated for comparison. The Lift values of six results are shown in Table 1. In the fixed-way result (Column3–7), we found that the negative datasets which have the same size with the positives got the highest value in top 1000 and top 2000, but it got low performance above 2000. The highest Lift values of fixed-size way from top 2000 to 10000 are obtained when the ratios of positives and negatives are set to be 1∶8, but they did not get good performance in other comparisons. The group-way got highest Lift value from top 1000 to top 8000 comparing with all fixed-size way. This is probably because one time size negative is too small to represent most of negatives. While more times negative datasets maybe too big, they can contain some positive PPI. Since the group-way which contains five different size negative datasets, so the results are not sensitive to the one exactly size. They can get a best performance. In brief, when we want to get robust performance, we repeated m times group-way to train models. In this identifying protein complexes task, we used five group-way to generate negative datasets as described in the paper.

**Table 1:** The average Lift value of fixed-size way and group-way for LPU model based on five-fold cross-validation^a^.

	Average	1∶1	1∶2	1∶4	1∶6	1∶8
Top 1000	***1371.13***	1362.2	1264.4	1201.92	1266.86	1240.84
Top 2000	***1081.43***	1018.77	981.95	961.13	1005.16	1011.48
Top 3000	***928.09***	846.15	831.28	825.95	862.29	891.54
Top 4000	***823.35***	738.74	727.59	735.4	753.78	797.27
Top 5000	***731.94***	656.37	648.71	663.14	664.51	713.5
Top 6000	***652.71***	592.41	588.44	599.6	600.04	645.76
Top 7000	***585.66***	537.16	538.86	548.63	545.86	582.98
Top 8000	***530.67***	493.21	496.37	500.46	501.37	529.81
Top 9000	482.87	454.23	459.31	461.84	463.18	***483.05***
Top 10000	441.72	421.79	425.88	429.11	428.05	***442.29***

Abbreviations: LPU, learning from positive and unlabeled data.

^a^ The numbers in bold and italic are the highest value in each evaluation.

### Performances of Protein Complexes Detection Methods

**Figure 3:**
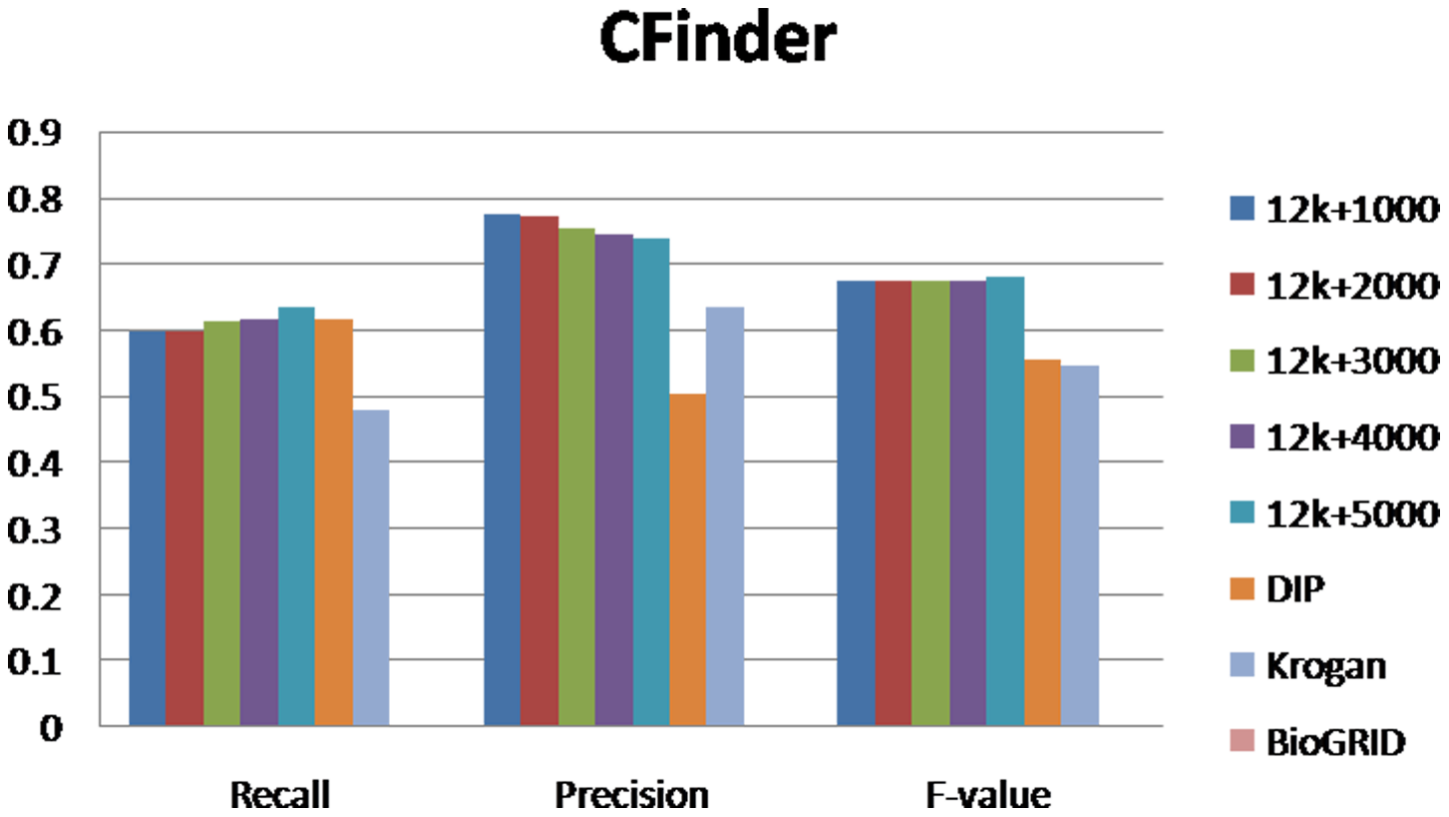
Performances of CFinder based on different protein-protein interaction networks. The Recall, Precision and F-value of CFinder based on DIP, BioGRID, Krogan and our selected reliable protein-protein interaction (PPI) network supplementing with top 1000, top 2000, top 3000, top 4000 and top 50000 predicted PPI.

**Figure 4:**
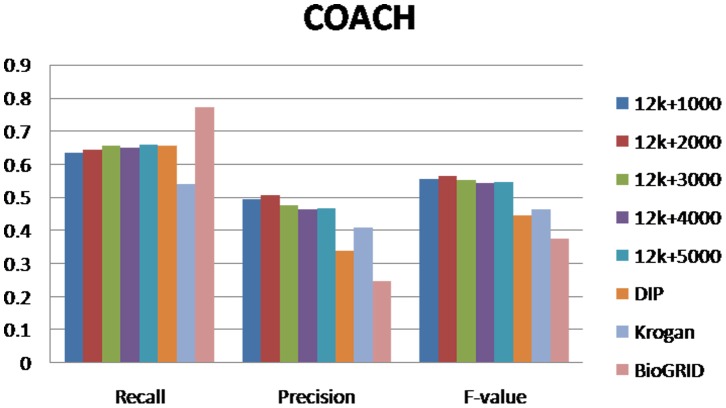
Performances of COACH based on different protein-protein interaction networks. The Recall, Precision and F-value of COACH based on DIP, BioGRID, Krogan and our selected reliable protein-protein interaction (PPI) network supplementing with top 1000, top 2000, top 3000, top 4000 and top 50000 predicted PPI.

**Figure 5:**
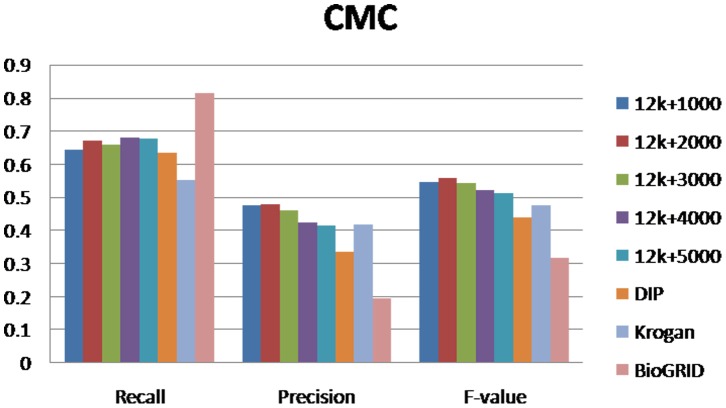
Performances of CMC based on different protein-protein interaction networks. The Recall, Precision and F-value of CMC based on DIP, BioGRID, Krogan and our selected reliable protein-protein interaction (PPI) network supplementing with top 1000, top 2000, top 3000, top 4000 and top 50000 predicted PPI.

**Figure 6:**
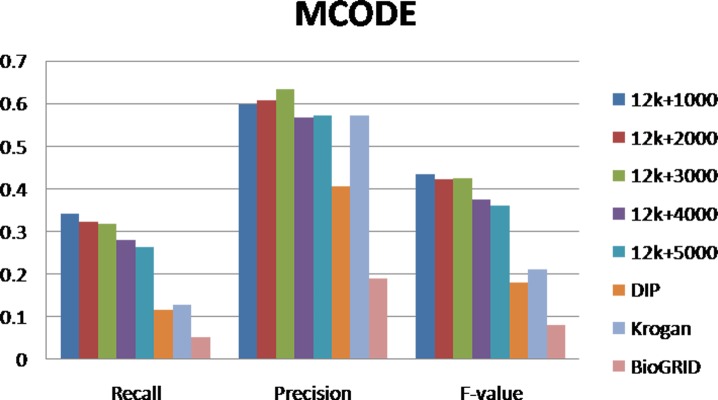
Performances of MCODE based on different protein-protein interaction networks. The Recall, Precision and F-value of MCODE based on DIP, BioGRID, Krogan and our selected reliable protein-protein interaction (PPI) network supplementing with top 1000, top 2000, top 3000, top 4000 and top 50000 predicted PPI.

**Figure 7:**
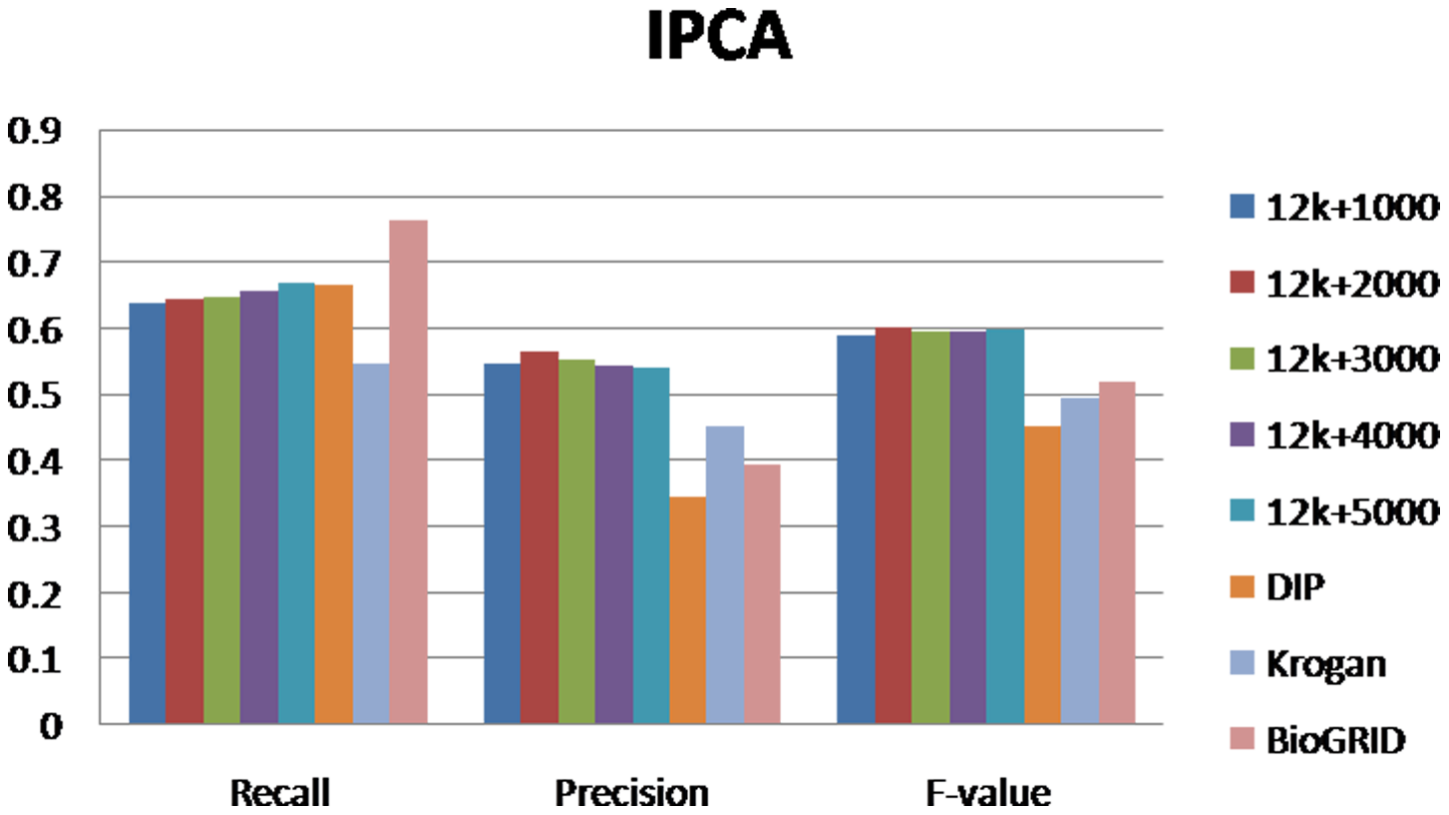
Performances of IPCA based on different protein-protein interaction networks. The Recall, Precision and F-value of IPCA based on DIP, BioGRID, Krogan and our selected reliable protein-protein interaction (PPI) network supplementing with top 1000, top 2000, top 3000, top 4000 and top 50000 predicted PPI.

**Figure 8:**
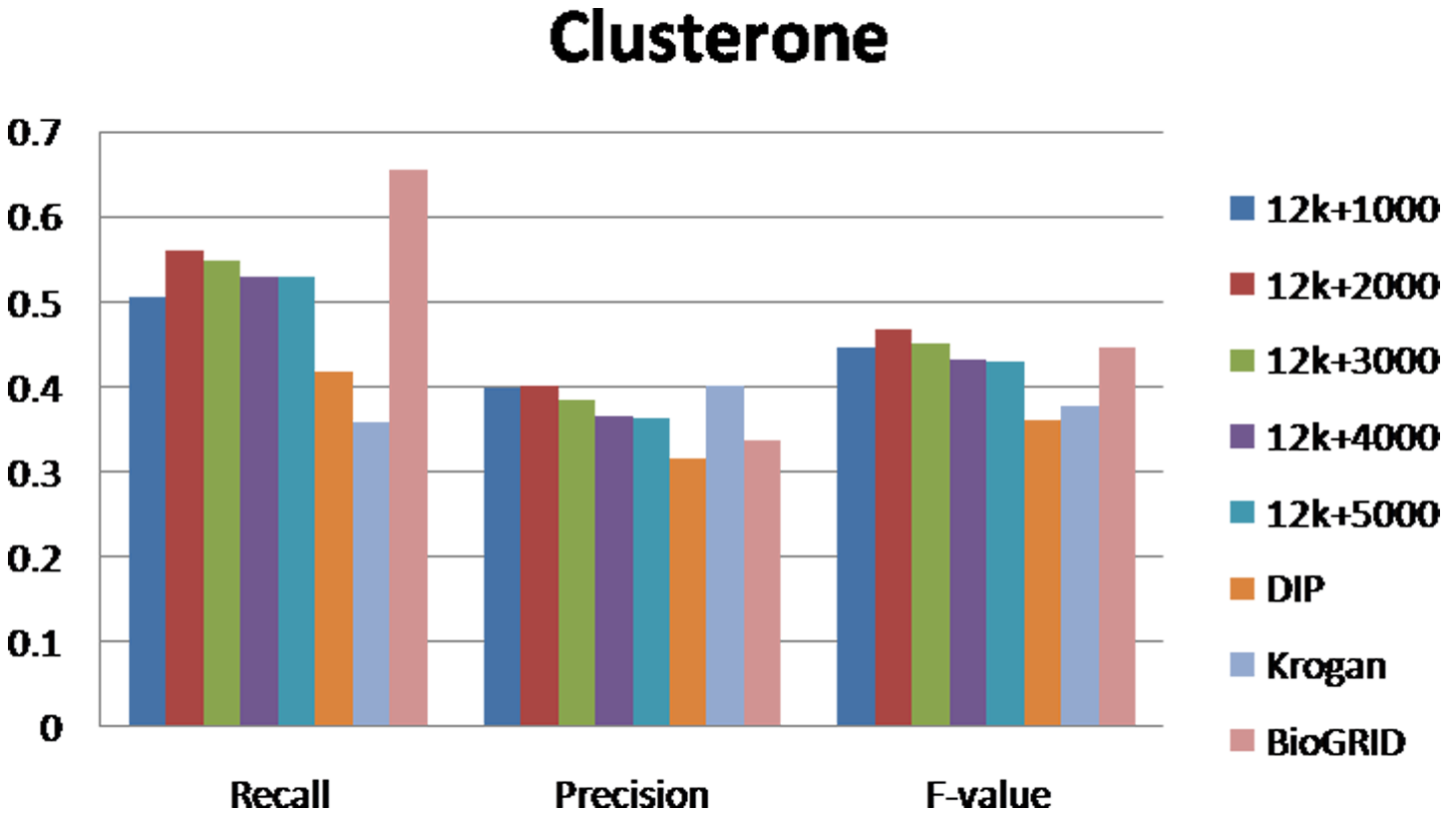
Performances of Clusterone based on different protein-protein interaction networks. The Recall, Precision and F-value of Clusterone based on DIP, BioGRID, Krogan and our selected reliable protein-protein interaction (PPI) network supplementing with top 1000, top 2000, top 3000, top 4000 and top 50000 predicted PPI.

For evaluating our method, we selected different size reconstructed networks for protein complexes detection and compared their performances with other existed popular PPI networks. Figure 3, 4, 5, 6, 7, 8, 9 show the performances of seven methods when selecting different networks (CFinder did not get results on BioGRID network in 48 hours). The F-value and precision of all these seven methods on our network are higher than on other existed popular networks. It indicates that many predicted PPI pairs are true PPI pairs and that incorporating them into the PPI network can improve protein complex identification. The recall based on our network is lower than BioGRID, this is probably because the BioGRID data set is very big and much more protein complexes can be detected from it. But the precision and F-value on BioGRID are very low. Meanwhile, we listed the best performances of each method achieved on our networks in Table 2. The highest F-value of COACH, IPCA, CMC, Clusterone, MCODE, CFinder and MCL achieved on our network are 0.565, 0.601, 0.559, 0.467, 0.434, 0.682 and 0.29, respectively. The highest Precision of COACH, IPCA, CMC, Clusterone, MCODE, CFinder and MCL achieved on our network are 0.505, 0.564, 0.478, 0.401, 0.598, 0.738 and 0.185, respectively.

**Figure 9:**
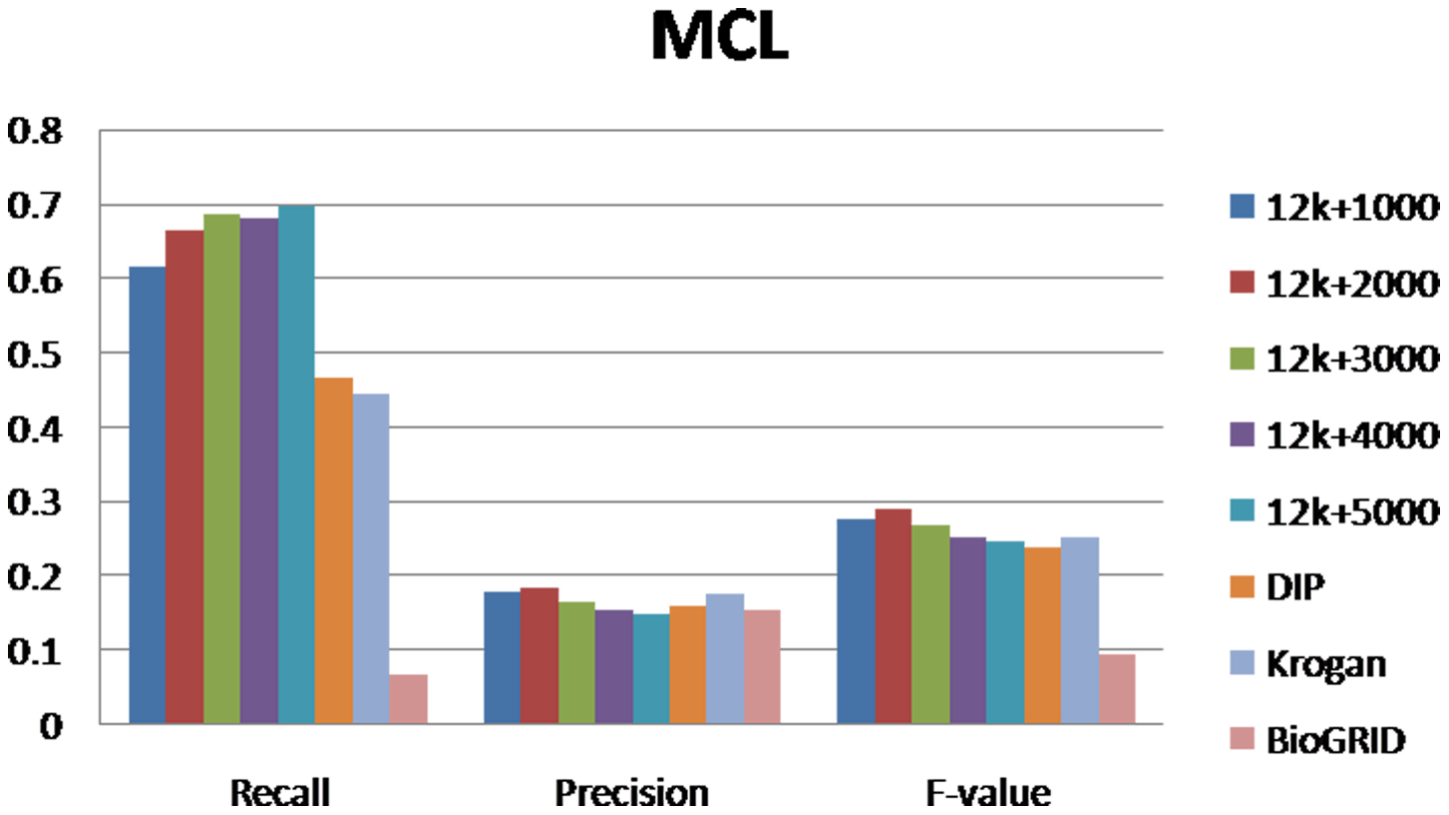
Performances of MCL based on different protein-protein interaction networks. The Recall, Precision and F-value of MCL based on DIP, BioGRID, Krogan and our selected reliable protein-protein interaction (PPI) network supplementing with top 1000, top 2000, top 3000, top 4000 and top 50000 predicted PPI.

**Table 2:** Performance Comparison Based on Reconstructed PPI Network (LPU), DIP, Krogan and BioGRID^a^.

Method	Evaluation	Ours	DIP	Krogan	BioGRID
COACH	F-Value	***0.565***	0.447	0.465	0.375
	Recall	0.642	***0.655***	0.539	0.772
	Precision	***0.505***	0.339	0.409	0.248
IPCA	F-Value	***0.601***	0.453	0.495	0.518
	Recall	0.642	0.664	0.547	***0.763***
	Precision	***0.564***	0.344	0.452	0.392
CMC	F-Value	***0.559***	0.438	0.476	0.317
	Recall	0.672	0.634	0.552	***0.815***
	Precision	***0.478***	0.335	0.419	0.196
Clusterone	F-Value	***0.467***	0.360	0.378	0.445
	Recall	0.560	0.418	0.358	***0.655***
	Precision	***0.401***	0.317	0.400	0.337
MCODE	F-Value	***0.434***	0.181	0.211	0.081
	Recall	***0.341***	0.116	0.129	0.052
	Precision	***0.598***	0.407	0.571	0.190
CFinder	F-Value	***0.682***	0.554	0.545	–
	Recall	***0.634***	0.616	0.478	–
	Precision	***0.738***	0.503	0.634	–
MCL	F-Value	***0.290***	0.237	0.252	0.095
	Recall	***0.664***	0.466	0.444	0.069
	Precision	***0.185***	0.159	0.176	0.154

Abbreviations: LPU, learning from positive and unlabeled data.

^a^ CFinder did not get results on BioGRID data within 48 hours. The numbers in bold and italic are the highest value in each evaluation.

Besides the above measurement, it also shows the same improvement in GO annotation analysis. We count the number of clusters with p-value [47] less than 0.01, a threshold which represents significant biological sense and compute the proportion of clusters which achieve low p-value. The proportion of clusters from various methods with low p-value are shown in Table 3. The biological significance of detected protein complexes from MCL is very low in all networks. This is probably because its detection method is only based on network structure without considering biology property. MCODE did not get a higher biological significance in CC (cellular components) on our networks. However, all the other methods achieved higher biological significance on our network than on the other 3 datasets. It indicates that protein complex identification algorithms achieve better performance when reconstructing PPI networks by combining PPI evidence from multiple sources.

**Table 3:** The Biology Significance Comparison Based on Reconstructed PPI Network (LPU), DIP, Krogan and BioGRID^a^.

Method	Evaluation	Ours	DIP	Krogan	BioGRID
COACH	MF	***0.509***	0.253	0.372	0.317
	BP	***0.570***	0.347	0.404	0.402
	CC	***0.362***	0.139	0.154	0.240
IPCA	MF	***0.588***	0.280	0.395	0.450
	BP	***0.646***	0.400	0.482	0.564
	CC	***0.492***	0.134	0.182	0.363
CMC	MF	***0.401***	0.197	0.295	0.141
	BP	***0.470***	0.239	0.280	0.160
	CC	***0.258***	0.087	0.096	0.087
Clusterone	MF	***0.244***	0.173	0.240	0.160
	BP	***0.277***	0.191	0.248	0.175
	CC	***0.150***	0.059	0.124	0.112
MCODE	MF	***0.411***	0.237	0.388	0.224
	BP	***0.383***	0.288	0.429	0.293
	CC	0.187	0.153	***0.265***	0.190
CFinder	MF	***0.668***	0.344	0.519	–
	BP	***0.726***	0.414	0.535	–
	CC	***0.541***	0.185	0.270	–
MCL	MF	0.094	0.097	***0.118***	0.044
	BP	0.098	0.105	***0.106***	0.066
	CC	***0.055***	0.049	0.042	0.044

Abbreviations: BP, biological processes; CC, cellular components; MF, molecular function;

^a^ CFinder did not get results on BioGRID data within 48 hours. The numbers in bold and italic are the highest value in each evaluation.

### Performances of Reconstructed Networks

We also evaluated our predicted PPIs through statistical analysis based on GO annotation. Since interacting proteins are likely involved in similar biological processes, they are expected to have similar functional annotations in gene ontology. Therefore, we measure the functional relevance between any pair of proteins that are connected by an edge using the semantic similarity between the GO terms annotated with the proteins, using a popular method [23]. Results shown the proportion of protein pairs in the PPI network whose similarity is above 0.5 in three branch of GO (BP, CC, MF) (Table 4). As the number of selected PPI increase, the relevance decrease slightly in BP and MF. But they are still higher than PPI in DIP, Krogan and BioGRID. We also measured the Pearson correlation coefficient between the gene expression profiles of every pair of genes, using Gene Expression Omnibus (accessed September 2011) data. We calculated proportion of protein pairs whose value is above 0.5 for each network. Results show that the PPI in our network are more functional relevance than other networks. All these indicate that our network not only have similar functions, but also have highly coexpressed. We gave a list of our predicted PPI networks in our website: http://202.118.75.18∶8080/PPINPredictor/.

**Table 4:** The comparison of protein pairs relevance in DIP, BioGRID, Krogan and our reconstructed networks supplementing with top 2000, 4000, 6000, 8000 and 10000 predicted PPI^a^.

	GO_CC	GO_BP	GO_MF	Co-express
12k+1000	***0.893***	***0.896***	***0.66***	***0.988***
12k+2000	0.889	0.892	0.657	***0.988***
12k+3000	0.884	0.889	0.653	***0.988***
12k+4000	0.879	0.884	0.648	0.987
12k+5000	0.876	0.88	0.643	0.987
DIP	0.791	0.741	0.541	0.962
Krogan	0.776	0.795	0.576	0.935
BioGRID	0.782	0.817	0.594	0.932

Abbreviations: GO, Gene Ontology; BP, biological processes; CC, cellular components; LPU, learning from positive and unlabeled data; MF, molecular function;

^a^ The numbers in bold and italic are the highest value in each evaluation.

In summary, our method gets a higher quality network for protein complexes detection. This illustrates that our approach of integrating PPI evidence from multiple sources is effective in protein complexes detection. These sources include different types of information, so it is more comprehensive than the existing methods that only consider GO ontology or DDI information. The integration of PPI information from multiple sources enables us to obtain more PPI pairs and enhance true PPI information. From our selected reliable positive PPI supplementing with predicted PPI, it addresses the false negative and false positive problem in the existing PPI network, hence improves the performance for protein complex identification. We plan to evaluate the contribution of each individual source towards protein complex identification in the future.

## Conclusion

We have integrated PPI information from multiple sources into protein complex identification. The evaluation of our method indicates that incorporating PPI information sources significantly improves the performance of protein complex identification algorithms. Future work includes evaluation of the contribution of each individual source toward protein complex identification. Additionally, we plan to incorporate additional features such as high-level structure information into the protein complex prediction task.
